# Identifying Children at Readmission Risk: At-Admission versus Traditional At-Discharge Readmission Prediction Model

**DOI:** 10.3390/healthcare9101334

**Published:** 2021-10-07

**Authors:** Hasan Symum, José Zayas-Castro

**Affiliations:** Industrial and Management Systems Engineering, University of South Florida, Tampa, FL 33620, USA; josezaya@usf.edu

**Keywords:** readmission, machine learning, pediatrics

## Abstract

The timing of 30-day pediatric readmissions is skewed with approximately 40% of the incidents occurring within the first week of hospital discharges. The skewed readmission time distribution coupled with delay in health information exchange among healthcare providers might offer a limited time to devise a comprehensive intervention plan. However, pediatric readmission studies are thus far limited to the development of the prediction model after hospital discharges. In this study, we proposed a novel pediatric readmission prediction model at the time of hospital admission which can improve the high-risk patient selection process. We also compared proposed models with the standard at-discharge readmission prediction model. Using the Hospital Cost and Utilization Project database, this prognostic study included pediatric hospital discharges in Florida from January 2016 through September 2017. Four machine learning algorithms—logistic regression with backward stepwise selection, decision tree, Support Vector machines (SVM) with the polynomial kernel, and Gradient Boosting—were developed for at-admission and at-discharge models using a recursive feature elimination technique with a repeated cross-validation process. The performance of the at-admission and at-discharge model was measured by the area under the curve. The performance of the at-admission model was comparable with the at-discharge model for all four algorithms. SVM with Polynomial Kernel algorithms outperformed all other algorithms for at-admission and at-discharge models. Important features associated with increased readmission risk varied widely across the type of prediction model and were mostly related to patients’ demographics, social determinates, clinical factors, and hospital characteristics. Proposed at-admission readmission risk decision support model could help hospitals and providers with additional time for intervention planning, particularly for those targeting social determinants of children’s overall health.

## 1. Introduction

Unplanned hospital readmissions disrupt the daily routine lives of patient and families, and expose patient to the risk of hospital-acquired infections and other potentially harmful conditions [1,2,3]. The preventable 30-day readmission rate has become a critical metric in assessing patient hospital care quality for hospitals and other healthcare providers [4]. Unplanned hospital readmissions are costly and often associated with adverse health outcomes and therefore have become a major policy concern [3,5,6]. In 2016, national estimates by the Agency for Healthcare Research and Quality (AHRQ), readmissions within 30 days resulted in hospital cost of $2.5 billion for children and $52.4 billion for adults [7]. To combat this, the Centers for Medicare and Medicaid Services (CMS) adopted the Hospital Readmissions Reduction Program (HRRP) in 2012 that penalizes hospitals with higher than expected readmission rates for targeted conditions and thus incentivizes hospitals to develop internal strategies to reduce readmissions [8]. Similarly, many states have begun imposing penalty payments to hospitals and Managed Care Organizations (MCO) with excess Medicaid readmission rates, particularly for pediatric readmissions [9,10]. These efforts resulted in a significant reduction in 30 days unplanned readmissions, particularly for targeted populations. Compared to adult and Medicare readmission rates, pediatric readmission rates remained unchanged, and one study even reported a national increment (8.2%) between 2010 and 2016 [11,12]. Consequently, readmission reduction efforts gained significant recent attention among healthcare providers and professionals in devising ways of reducing readmission risk among children [13,14]. An improved readmission prediction model could help hospitals and healthcare providers to identify high-risk patient groups and, implement interventions in timely manner that reduce the risk of unplanned readmission within days of discharges.

In the last decade, with the wide adoption of electronic medical record systems across hospitals and other provider systems, researchers have focused on predictive analytics to identify patient at greater risk of being readmitted and finding ways to prevent unplanned hospital visits [15,16,17]. Accurate and advanced readmission risk prediction would provide opportunities for hospitals and insurance companies to design and implement general or condition-specific interventions toward those who might need it most [18]. Owing to the CMS hospital readmission reduction programs, prediction of adult and Medicare patients have been the subject of substantial research and tackled by various hospital point-of-care approaches [19,20]. However, readmission prediction for children have received limited attention [21,22]. Prior pediatric readmission studies are thus far limited to the development of a prediction model after patient hospital discharges [23,24]. Most of these after-discharge readmission prediction studies reported predictive models for a 30-day readmission and, recently one study showed promise for 7-day pediatric readmission prediction [25,26,27]. However, these after-discharge predictive models might provide a limited amount of time for hospitals and providers to identify high-risk children and devise any appropriate general or patient intervention plans, mainly due to characteristics and timing of pediatric readmission. Timing of the prediction model application in pediatric hospital care is important for multifactorial reasons. First, the timing of the 30-day pediatric readmissions is positively skewed with approximately 40% of the incidents within the first week of hospital discharges [28]. Second, pediatric readmission prevention often requires multi-faceted programs including clinical and non-clinical interventions (e.g., targeting social determinants of health). These interventions often involve coordinated participation from various care providers, hospitals, and healthcare insurers (e.g., MCOs) [13]. The skewed readmission timing distribution coupled with the delay in health information exchange between healthcare providers might jeopardize multifactorial interventions plan due to limited time availability [29,30]. Consequently, strategies for reducing pediatric readmissions need to account for the higher frequency of readmissions within the first week of discharge, thus warranting an earlier but similar predictive readmission model to better target the high-risk patients. Therefore, it is crucial to develop an advanced readmission risk prediction model, which can be coupled more effectively with appropriate intervention programs to reduce readmission risk, and ultimately improve quality of care [31].

Our study hence aimed to develop prediction models that can better identify those children that are at high risk of unplanned hospital readmission visits. In this study, we propose a novel pediatric readmission prediction model at the time of hospital admission, which we hypothesized would have provided physicians and care providers with additional time for intervention planning, particularly for those targeting social determinants of children’s overall health. In addition, we looked into the predictability performance of our proposed model compared with the existing at discharge model, investigated the performance by diagnosis groups and the timing of implementing pediatric readmission models.

## 2. Materials and Methods

### 2.1. Study Setting

Using the Hospital Cost and Utilization Project (HCUP) State inpatient database, this retrospective study included all pediatric admissions from 1 January 2016 to 30 September 2017, across all Florida’s hospitals. Developed by the AHRQ, the HCUP SID is an all-payer dataset that includes the uninsured database of hospital inpatient stays across all non-federal hospitals [32]. The dataset contains patient-level information on demographic characteristics, insurance status, and International Classification of Diseases, 10th Revision, Clinical Modification (ICD-10-CM) diagnosis and procedure codes, patient location, and hospital charges of hospital visits from 265 acute care hospitals across 67 Florida counties. Data on hospitals, including geo-locations, were obtained from the American Hospital Association Guide. Data on community-level health determinants were derived from the American Community Survey (ACS) by linking patient ZIP codes through Uniform Data System (UDS) Mapper crosswalk [33]. We excluded adult patients (>18), residential addresses outside Florida, discharges against medical advice, and cases of in-hospital mortality from the dataset. Institutional review board approval was not required as determined by the local Institutional Review (IRB) Board.

### 2.2. Outcome Variable

The primary outcome was all-cause readmission to any Florida hospital within 30 days following discharge of an acute care hospitalization. We used the previously validated all-cause Pediatric All-Condition Readmission algorithm by the Boston Children Hospital to identify pediatric readmissions [34]. Consistent with the prior studies, only the first readmission within 30 days was considered and subsequent admissions after 30 days from discharge were identified as another index hospitalization [34,35]. Besides, our study considered only readmission events that occur in children younger than 18 years and excluded readmissions for planned procedures and chemotherapy similar to prior studies [36,37].

### 2.3. Predictors

**Figure 1:**
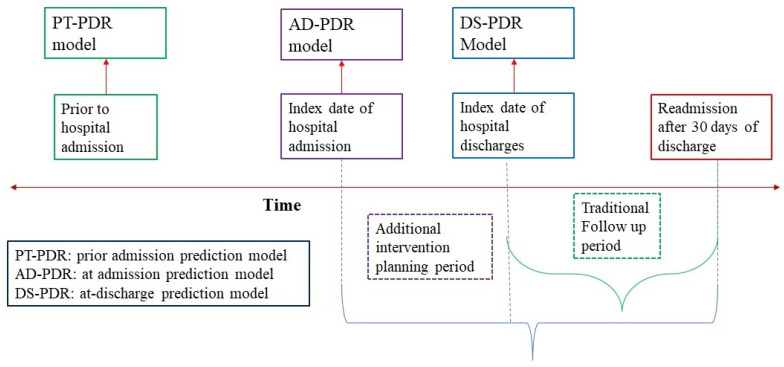
Readmission prediction model at different time points of care.

In this study, we evaluated the pediatric readmissions prediction model using patient information and available data for three major time points: (1) prediction model that uses data available at the time of hospital admission or transferring to another acute care hospital, (2) standard readmission prediction model that uses all available information during discharge time, and (3) hospital admission prediction model that uses only available patient information and social determinants of health before any hospitalization event occurs. Predictors for the three models—at-admission prediction model (AD-PDR), at-discharge prediction model (DS-PDR), and prior to the hospital admission prediction model (PT-PDR)—were included based on the availability of the information at that certain time point (Figure 1). PT-PDR models included patient demographics, socioeconomic status, provider density, prior hospital visit history, and community-level social determinants of health. At admission model (AD-PDR) included all variables included in the PT-PDR model, diagnosis presented at the time of hospital admission, and admitting hospitals detailed information. Finally, the conventional at-discharge prediction model (DS-PDR) includes all predictor variables from PT-PDR and AD-PDR models as well as diagnosis, hospital procedures, and discharge information. Table 1 shows the predictor variables included in three-readmission prediction models.

PT-PDR models included available variables before hospital admission including demographics, socioeconomic status, provider density, prior hospital visit, and community-level social determinants of health (Supplemental Table S1). Demographic variables included in our study were age (0–1, 1–5, 5–8, 8–12, and ≥12), race (African American, White, Hispanic, and others), and gender. Patients’ insurance status (public fee for service, Medicaid managed care, private, and uninsured) and community-level social determinants of health were used as proxy measures of individual and neighborhood socioeconomic conditions [38,39]. Provider density was considered as a binary variable (high/low), low provider density is considered for if patients live in the designated medically underserved area (MUA) and counties. Designated MUA status was determined using the U.S. Health Resources and Services Administration (HRSA) classification [40]. History of patient’s hospital visits including unplanned treat-and-release emergency department (ED) visits and readmissions within one year of index admissions were also included in our analysis. Social determinants of health (SDH) variables considered in our study were the percentage of people with an income below 100% federal poverty level (FPL), the percentage of homes with no vehicles, the percentage of people with no high school diploma, and the percentage of the unemployed person. These community-level SDH variables affected hospital visit behaviors reported in prior studies and included in our study at the ZIP code tabulation area (ZCTA) level, a generalized area representation of the ZIP codes used by the U.S. Census [41].

The AD-PDR models included admitting hospitals characteristics, patient travel information, individual-level social determinants of health, diagnosis presented at the time of hospital admission, and the variables included in the PT-PDR model. Hospital-level covariates are children’s hospital status, location (division, metropolitan, and micro/rural), ownership status (for-profit and non-profit/government), and hospital bed size (large, medium, and small). Travel distances between patients’ residences and discharge hospitals were calculated by geocoding using geographical information software (ArcGIS 10; Ersi Inc., Redlands, CA, USA). The individual-level SDH variable was a binary variable indicating potential health hazards related to children’s family conditions (e.g., housing and parent instability). The ICD-10-CM admitting diagnosis codes used to characterize hospital visits by patient disease complexity for at admission, AD-PDR models. Each of the admitting diagnosis ICD-10-CM codes is included as a binary (yes/no) variable included in the building AD-PDR models.

The DS-PDR models included patient discharge information, ICD-10 CM diagnosis, and procedures along with variables used in the PT-PDR and AD-PDR models. The ICD-10 CM diagnosis and procedures codes during hospitalization were coded into individual binary codes (yes/no) and included in the model. The other patient-level factors included only in the DS-PDR model were inpatient LOS (0–3, 3–8, and ≥8 days), and discharge planning (routine, post-acute facility, and home health care). The total number of variables included in building the prediction model for PT-PDR, AD-PDR, and DS-PDR was 18, 3721, and 6324, respectively.

### 2.4. Modeling and Analysis

**Figure 2:**
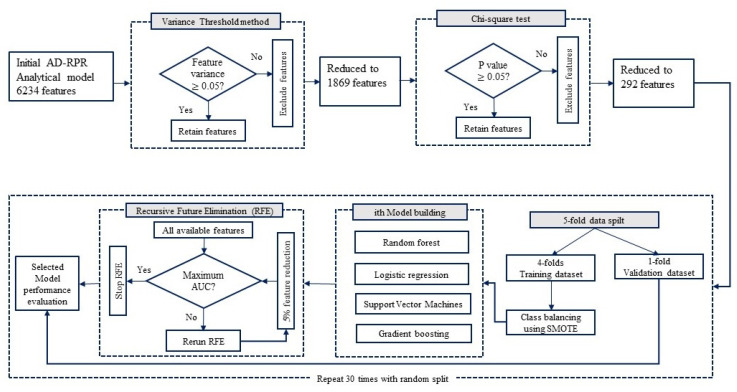
Model building and performance evaluation process.

The overall model training and validation process we followed is shown in Figure 2. The overall missing data rate was <0.5%, which we imputed using multiple regression chained equations. We initiated our feature selection process with the variance threshold method, which eliminates features with certain threshold values. We examined the overall distribution of our features and selected a cut-off point of 0.05 for selecting the features. After the variance threshold method, we implemented a chi-square feature selection model with a 0.05 level of significance. The Chi-square feature selection process is computationally efficient and has been widely adopted in prior feature selection research studies [42]. We then used a recursive feature elimination (RFE) technique with a repeated fivefold cross-validation process to evaluate the performance of the prediction model. The RFE approach trained all the available variables and assign a relative weight for the developed prediction model. Therefore, we can eliminate unimportant features by assigning a cutoff weight value. In our case, we set the cut-off value 5% after each iteration, which eliminates 5% of the total number of low weighted features iteratively, until we have the maximum performance metric.

For the repeated cross-validation process, each cohort (e.g., at-discharge AD-PDR) entire dataset was divided into 5 equal cross-validation folds. For each cross-validation repetition, each fold is alternatively used as the test dataset while training our predictors on the other remaining folds. The hyperparameter of each technique was optimized through a grid search with 10 repeated 5-fold cross-validation iterations. While training, we also explored the issues with class imbalanced problems by using the Synthetic Minority Over-sampling Technique (SMOTE) on the training dataset [43,44]. We repeated the cross-validation process 30 times on each cohort to obtain the average performance of each learning model.

We developed and investigated several established machine learning algorithms for each model cohort to identify children at high risk for 30-day unplanned readmission. The target variables for the three patient cohorts based on timing was binary variable (yes/no) if the children have been readmitted within 30 days of hospital discharge. To assesses the performance of the learning model, we applied logistic regression (LR) with backward stepwise selection, Decision tree (C4.5), Support Vector machines (SVM) with the polynomial kernel, and Gradient Boosting (GB) algorithms for each disease cohort. The area under the receiver operating characteristics (ROC) curve (AUC) was used to evaluate the performance of each prediction model. The average AUC values from traditional DS-PDR models were considered as baselines for the performance comparison of the machine-learning algorithms. All statistical analyses were performed using R studio, and a two-sided *p*-value less than 0.05 was considered statistically significant.

## 3. Results

The analysis included 87,865 index hospital visits by 64,597 children with a mean age of 7.8 years with 50.04% females from 1 January 2016 to 30 September 2017. Among these index visits, we identified 7288 (8.29%) pediatric hospital 30-day readmission visits. The baseline patient demographics and hospital characteristics for the hospital visits were provided in Table 2. The distribution of the timing of 30-days readmission is illustrated in Supplemental Figure S1. We found from our initial analysis that 35.5% of hospital readmissions occurred within the first seven days of hospital discharge, where the highest percentage of readmission occurred on day 3. Out of the total of 87,865 hospital visits, 14,849 (16.9%) visits had a different primary diagnosis at discharge time than admitting diagnosis. This dissimilarity between discharge primary diagnosis and admitting diagnosis occurred for the children hospital visits with admitting diagnosis (Supplemental Figure S2) associated with mental health disorder (26.1%); respiratory system disease (7.3%); Injury and poisoning (6.3%); and Symptoms, signs, and ill-defined conditions (53.2%).

### 3.1. Prediction Performance Comparison

Table 3 summarizes the comparative performance of four learning algorithms for three different prediction models. The performance of the at-admission (AD-PDR) model was comparable with the at-discharge (DS-PDR) model for all four algorithms. Prior unplanned admission prediction (PT-PDR) models showed the lowest average AUC for all four prediction models. SVM with Polynomial Kernel algorithms outperformed all other algorithms for AD-PDR and DS-PDR models, while in PT-PDR the Gradient Boosting model outperforms other algorithms. Among all four algorithms, RF models showed the lowest average AUC for all three prediction models.

### 3.2. Important Features of Pediatric Readmissions

We also extracted important features from the Random Forest algorithms for all three prediction models. The top 10 important features are shown in Table 4. Important features associated with increased readmission risk varied widely across the type of prediction model and were mostly related to patients’ demographics, SDHs, clinical factors, and hospital characteristics. The history of the prior hospital visits was most important for the PT-PDR model and the second most important feature for both AD-PDR and DS-PDR models. The higher the accumulated times a child has been hospitalized, the more likely the patient will be readmitted after hospital discharge. The low healthcare provider density was found an important factor for the three readmission prediction models. Discharges to post-acute facilities and longer travel distances were also found within the top ten important features for both the at-admission and at-discharge models. Children’s insurance status with Public Managed Care was found within the top five most weighted features for PT-PDR and AD-PDR models. African American children, children aged 5 to 8, and adolescent children were significantly associated with increased readmission risk for only PT-PDR models.

The presence of comorbidity and complex procedures were also important predictors of readmission for AD-PDR and DS-PDR. Disruptive mood disorder was the most important feature for both AD-PDR and DS-PDR models. The other important clinical features for the AD-PDR model were Dehydration and Abdominal pain. For the DS-PDR model, Pneumonia and Major Depressive Disorder-recurrent were other top ten clinical diagnoses related to high-risk readmission. The Drainage of the Spinal Canal and Resection of the Appendix procedure was also found important for predicting readmission at the DS-PDR model. Children’s hospital status was only found important for the AD-PDR model and longer hospital stay was found within the top ten features for DS-PDR models. Children living with challenging family conditions and in poor neighborhoods were found as important features for both PT-PDR and AD-PDR models. Similarly, children living in communities with fewer high school diplomas and a higher percentage of unemployed persons were found important for the PT-PDR models.

## 4. Discussions

In summary, we developed and compared several variants of machine learning-based predictive models for three different care timepoints that can improve the prediction of pediatric readmission, with the possibility of at-admission pediatric readmission risk prediction. To our knowledge, this is the first study to develop an at-admission pediatric readmission model and compared prediction performance with the traditional at-discharge readmission prediction model. Our proposed at-admission all-causes readmission prediction model showed similar prediction performance compared with the at-discharge model. In terms of predictive power, the models we developed showed comparable results with other published works [27,28,30,45,46]. However, these models considered all-condition or all-surgeries 30-day readmission during hospital discharge time point, therefore lacks the ability to an equivalent comparison of these models to our proposed DS-PDR models. Therefore, this study highlights the potential of the AD-PDR model in identifying high-risk children during hospital admission over traditional at-discharge approaches.

The pre-admission model in our study showed lower discrimination compared with at admission and at-discharge readmission models mainly due to lack of patient-level clinical information. Therefore, a single pre-admission model may not be sufficient in a clinical setting for identifying high readmission risk children. The similar performance of the AD-PDR models and DS-PDR models reported in this study suggests that pediatric readmission risk prediction during both at-discharge and at-hospital admission can be used by hospital providers to design and implement appropriate intervention programs. This additional time provided by the at-admission readmission risk prediction (AD-PDR) model could allow comprehensive care transition and discharge planning particularly for high-risk returning patients [31,47]. Although the AD-PDR model allows pediatric readmission prediction, the model potentially misclassifies certain patient populations, mainly due to a lack of adequate diagnosis data. This misclassification of the AD-PDR model is likely related to the patients admitted for unclear admitting diagnosis (e.g., unspecified fever and abdominal pain), as their primary diagnosis usually (53.2% reported in our study) changed after additional clinical tests. Besides, predicting readmission for children with unspecified mental health disorders is challenging due to the unpredictable nature of the episodes [48].

In our study, we found a variation of extracted important features across three readmission prediction models. History of prior hospital admissions, medical complexity, and non-acute post-discharge were found important features in predicting readmission, which is consistent with the previous investigations [22,24,28,49]. Although these factors are not easily modifiable for most conditions, comprehensive intervention strategies including better discharge planning (e.g., telephone call) and care coordination can mitigate the risk of pediatric readmission [13,31,50,51]. The findings of prior hospital visits in all three readmission models suggest that there might exist an unresolved system issue associated with the quality and clarity of discharge education and access to pediatric care for a certain patient population [36]. Besides, living in medically underserved communities as important factors suggests residents in these areas may have limited access to pediatric care due to geographical location [52,53]. This unequal access to care might result from a combined effect with persistent rural–urban disparities in pediatric care access and a high degree of pediatric care regionalization [53,54,55]. These findings highlight to policymakers the need to develop a tailored interventions/programs particularly, for these MAU areas ensuring necessary pediatric care access. Besides, several important community-level SDHs features (e.g., high school graduation and employment rate) found in our study in readmission prediction suggest an existing disparity in pediatric care, mainly due to socioeconomic inequality [56,57]. Moreover, longer travel distance as an important factor suggests the persistence of rural-urban disparities in children’s healthcare due to the high regionalization of pediatric care. Therefore, interventions including components that are implemented before (e.g., parent education and health literacy) and post-discharge (e.g., need-based assistance programs and discharge follow-up appointments) can help to mitigate readmission risk across the vulnerable population [58].

This study has several common limitations, most of which are related to a retrospective analysis of administrative claim databases. First, the HCUP database is an administrative claim dataset that uses ICD codes to classify patients’ medical diagnoses, procedures, and outcomes. The possibility of coding inaccuracy or incorrect information cannot be dismissed. Second, although our study makes a significant contribution of presenting an at-admission readmission prediction model across Florida hospitals, the findings of this study may not be generalizable to other U.S. states or international countries’ patient populations. Third, our research did not include information regarding laboratory tests, patient detailed vitals, parent health literacy, and post-acute care quality, thus, including these factors may have improved prediction performance. Fourth, the dataset does not include data from federal hospitals (e.g., Veterans Affairs Hospital) or out-of-state hospital deaths or readmissions after the initial index admission. However, pediatric admissions in federal hospitals are few compared to non-Federal hospitals. In addition, out-of-state pediatric readmissions are expected to have minimal impact on our results, due to the unique geographical location of Florida State. Finally, we include community-level SDHs in the ZTCAs level, and more precise census tract level data or patient-provided information may have improved accuracy in capturing community-level variables.

## 5. Conclusions

Although pediatric readmissions are costly and rates have increased over time, accurate prediction of high readmission risk children and timely intervention implementation remains challenging for the care providers. To explore the possibility of improving pediatric readmission risk prediction, a novel at-admission readmission prediction model was proposed and compared with the traditional at-discharge models. Similar performance achieved by the proposed at-admission models suggests potential in early identification of high-readmission risk children without trading off the prediction performance over traditional at-discharge approaches. This additional intervention planning time could allow hospitals and payers (e.g., managed care providers) to devise comprehensive care transition (e.g., follow-up call), discharge planning, and non-clinical interventions (e.g., targeting social determinants) particularly across the vulnerable population. In addition, the findings of non-clinical important factors such as living in a disadvantaged community and longer travel distance other than the clinical risk factors imply the existence of rural–urban and socioeconomic disparities in pediatric care access. Therefore, these findings reinforce the need to develop a tailored interventions/programs by policy-makers particularly, for these underserved areas.

## Figures and Tables

**Table 1:** Predictor variables for the three pediatric readmission prediction models.

Variable Type	Prediction Model Prior to Admission (PT-PDR)	Prediction Model at Admission (AD-PDR)	Prediction Model at Hospital Discharge (DS-PDR)
Demographics	X	X	X
Socioeconomic status	X	X	X
Provider density	X	X	X
History of hospital visits	X	X	X
Community-level social determinants of health	X	X	X
Individual-level social determinants of health		X	X
Diagnosis at admission		X	X
Hospital characteristics		X	X
Hospital travel distance		X	X
Diagnosis during hospitalization			X
Hospital procedures			X
Discharge planning			X
Hospital length of stay			X

**Table 2:** Patient and hospital characteristics at 30-day pediatric readmissions.

Variable	Total (N = 87,865) n (%)	No Readmission (N = 80,577) n (%)	Readmission (N = 7288) n (%)	*p* Value
Age (y)				
0–1	12,321 (14.0)	11,502 (14.3)	819 (11.2)	<0.001
1–5	14,624 (16.6)	13,361 (16.6)	1263 (17.3)	
5–8	9020 (10.3)	8344 (10.4)	676 (9.3)	
8–12	11,856 (13.5)	10,820 (13.4)	1036 (14.2)	
12–17	40,044 (45.6)	36,550 (45.4)	3494 (47.9)	
Gender				
Male	43,882 (49.9)	40,141 (49.8)	3741 (51.3)	0.21
Female	43,983 (50.1)	40,436 (50.2)	3547 (48.7)	
Race				
White	34,367 (39.1)	31,503 (39.1)	2864 (39.3)	<0.01
African American	26,676 (30.4)	24,251 (30.1)	2425 (33.3)	
Hispanic/Latin	23,079 (26.3)	21,341 (26.5)	1738 (23.8)	
Others	3743 (4.3)	3482 (4.3)	261 (3.6)	
Insurance				
Public FFS	10,385 (11.8)	9132 (11.3)	1253 (17.2)	<0.01
Medicaid MCO	51,928 (59.1)	47,830 (59.4)	4098 (56.2)	
Private	20,007 (22.8)	18,513 (23.0)	1494 (20.5)	
Uninsured	5545 (6.3)	5102 (6.3)	443 (6.1)	
Travel distance (home to index hospital)				
<10 miles	14,435 (35.1)	12,561 (35.3)	1874 (33.5)	<0.001
10–20 miles	11,874 (28.9)	10,437 (29.4)	1437 (25.7)	
≥20 miles	14,798 (34.0)	12,524 (35.3)	2274 (40.8)	
Discharge disposition				
Routine	35,063 (85.3)	32,890 (92.5)	2173 (38.9)	<0.001
Post–acute Facility	4344(10.6)	1003 (2.8)	3341 (59.8)	
Home Health care	1700 (4.1)	1629 (4.7)	71 (1.3)	
Length of stay				
0–3 days	35,374 (40.3)	32,907 (40.8)	2467 (33.9)	<0.001
3–8 days	24,368 (27.7)	22,386 (27.8)	1982 (27.2)	
≥8 days	28,123 (32.0)	25,284 (31.4)	2839 (39.0)	
Hospital type				
Children	11,513 (13.1)	10,203 (12.7)	1310 (18.0)	<0.001
Adult	76,352 (86.9)	70,374 (87.3)	5978 (82.0)	
Hospital location				
Metro	87,447 (99.5)	80,167 (99.5)	7280 (99.9)	<0.001
Micro/Rural	418 (0.05)	410 (0.05)	8 (0.01)	
Hospital ownership				
Non-profit/Government	15,713 (17.9)	14,897 (18.5)	816 (11.2)	<0.001
For profit	72,152 (82.1)	65,680 (81.5)	6472 (88.5)	
Hospital Size				
Large	56,628 (64.4)	51,684 (64.1)	4944 (67.8)	<0.001
Medium	26,722 (30.4)	24,679 (30.6)	2043 (28.0)	
Small	4515 (5.1)	4214 (5.2)	301 (4.1)	

**Table 3:** AUC performance comparison of the three readmission prediction models.

Machine Learning Algorithms	Prediction before Admission (PT–PDR) (AUC, 95% CI)	Prediction at Admission (AD–PDR) (AUC, 95% CI)	Prediction at Discharge (DS–PDR) (AUC, 95% CI)
Support Vector Machines with Polynomial Kernel	0.57 (0.54–0.60)	0.68 (0.66–0.70)	0.73 (0.70–0.76)
Logistic regression	0.59 (0.56–0.62)	0.65 (0.62–0.68)	0.69 (0.66–0.72)
Gradient Boosting	0.60 (0.57–0.63)	0.66 (0.64–0.68)	0.67 (0.63–0.71)
Random Forest	0.56 (0.51–0.61)	0.61 (0.57–0.65)	0.64 (0.60–0.68)

**Table 4:** Important features extracted by support vector machines with polynomial algorithm.

Rank	Features in PT-PDR Model (Weight)	Features in AD-PDR Model (Weight)	Features in DS-PDR Model (Weight)
1	Prior hospital visit (0.31)	Disruptive mood disorder (0.24)	Disruptive mood disorder (0.16)
2	Age (12–17) (0.09)	Prior hospital visit (0.11)	Prior hospital visit (0.10)
3	Provider density (0.08)	Dehydration (0.09)	Pneumonia (0.08)
4	Public Managed Care (0.06)	Abdominal pain (0.06)	Major Depressive Disorder- recurrent (0.08)
5	African American (0.05)	Public Managed Care (0.05)	Drainage of Spinal Canal (0.06)
6	% of people with an income below 100 FPL (0.4)	Provider density (0.05)	Length of stay (0.05)
7	% of people with no high school diploma (0.04)	Post-acute facility (0.05)	Resection of Appendix (0.03)
8	Age (5–8) (0.04)	Hospital travel distance (0.02)	Post-acute facility (0.03)
9	% of the unemployed person (0.03)	% of people with an income below 100 federal poverty level (0.02)	Provider density (0.03)
10	% of homes with no vehicles (0.02)	Children Hospital (0.02)	Hospital travel distance (0.02)

## Data Availability

In this research, limited datasets were used, and datasets are available through the Healthcare Cost and Utilization Project (HCUP). Website: https://www.hcup-us.ahrq.gov/db/state/siddbdocumentation.jsp (accessed on 3 April 2019).

## Supplementary Materials

The following are available online at https://www.mdpi.com/article/10.3390/healthcare9101334/s1, Figure S1: Timing distribution (%) of pediatric readmission after hospital discharge, Figure S2: Changes (%) in primary diagnosis after hospital admission by diagnosis groups, Table S1: Predictor variables included in the readmission prediction models.
