# Supplementary material for: Identifying Children at Readmission Risk: At-Admission versus Traditional At-Discharge Readmission Prediction Model
Source: Healthcare (Basel). 2021 Oct 7;9(10):1334. doi: 10.3390/healthcare9101334 (PMC8544577; doi:10.3390/healthcare9101334)
Supplement: Supplementary file 1 [file healthcare-09-01334-s001.zip › healthcare-1370035-supplementary.pdf]

**Table S1:** Predictor variables included in the readmission prediction models.

| Variable Type | Prediction Model Prior to Admission (PT-PDR) | Prediction Model at Admission (AD-PDR) | Prediction Model at Hospital Discharge (DS-PDR) |
|---|---|---|---|
| **Demographics** | X | X | X |
| Age | | | |
| Race | | | |
| Gender | | | |
| **Socioeconomic status** | X | X | X |
| Insurance type | | | |
| **Provider density** | X | X | X |
| Designated medically underserved area (MUA) | | | |
| **History of hospital visits** | X | X | X |
| Prior Treat-and-Release ED visits | | | |
| Prior hospital readmission | | | |
| **Community-level social determinants of health** | X | X | X |
| The percentage of people with an income below 100% federal poverty level (FPL) | | | |
| The percentage of homes with no vehicles | | | |
| The percentage of people with no high school diploma | | | |
| The percentage of the unemployed person | | | |
| **Individual-level social determinants of health** | | X | X |
| Potential health hazards related to children's family conditions | | | |
| **Diagnosis at admission** | | X | X |
| **Hospital characteristics** | | X | X |
| Children's hospital status | | | |
| Hosptial location | | | |
| Hospital ownership type | | | |
| Hospital bed size | | | |
| **Hospital travel distance** | | X | X |

| | |
|---|---|
| **Diagnosis during hospitalization** | X |
| **Hospital procedures** | X |
| **Discharge planning** | X |
| **Hospital length of stay** | X |

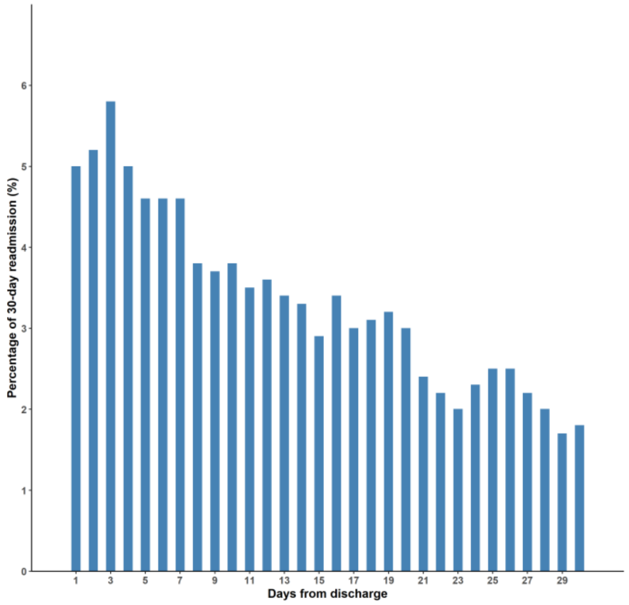


**Figure S1:** Timing distribution (%) of pediatric readmission after hospital discharge.

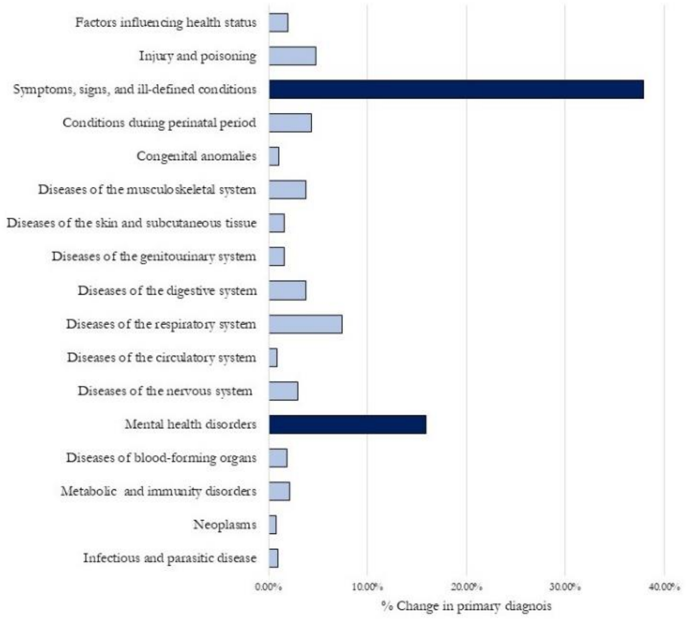


**Figure S2:** Changes (%) in primary diagnosis after hospital admission by diagnosis groups.
